# Successful dissolution of a giant bezoar after enzyme complex therapy

**DOI:** 10.23938/ASSN.1154

**Published:** 2026-03-09

**Authors:** Lucas Sabatella, Juan José Gascón, Nuria Blanco, Fernando Rotellar, Víctor Valentí

**Affiliations:** Department of Surgery Clínica Universidad de Navarra Pamplona Navarra Spain

**Keywords:** Bezoars, Intestinal Pseudo-Obstruction, Enzymes and Coenzymes, Stomach, Bezoares, Seudoobstrucción Intestinal, Enzimas y Coenzimas, Estómago

## Abstract

Bezoars are an uncommon cause of intestinal obstruction and may be managed using several approaches. Because recurrence can occur, invasive or surgical treatments should not be considered first-time when conservative options are feasible.

We report the case of a 67-year-old man with a previously asymptomatic giant gastric phytobezoar who developed subocclusive symptoms. A review of the literature was performed to evaluate available treatment strategies, as no standard therapy exists to date for giant bezoars. A conservative approach was selected using an oral enzyme complex (Digeston Plus®) containing digestive enzymes and probiotic bacteria. The patient completed a three-month treatment course, leading to symptom resolution and near-complete dissolution of the bezoar without the need of invasive intervention.

## RESUMEN

Los bezoares son una causa poco frecuente de obstrucción intestinal y pueden tratarse mediante diversos enfoques. Dada la probabilidad de recidiva, los tratamientos invasivos o quirúrgicos no deben considerarse de inicio.

Presentamos el caso de un hombre de 67 años con antecedentes de un fitobezoar gástrico gigante asintomático, que comenzó a desarrollar síntomas suboclusivos. Se realizó una revisión de la literatura para resumir los enfoques terapéuticos disponibles para los bezoares gigantes, para los cuales no existen tratamientos de referencia establecidos. Se decidió entonces optar por un tratamiento conservador utilizando un complejo enzimático (Digeston Plus®), que incluye enzimas digestivas y bacterias probióticas. El paciente siguió el tratamiento durante tres meses, lograndose la resolución de los síntomas y la desaparición casi completa del bezoar.

## INTRODUCTION

A bezoar is a conglomerate of indigestible material that accumulates within the gastrointestinal tract^1^. These masses may result from the ingestion of substances that cannot be digested, whether consumed intentionally or accidentally^2^. Although bezoars can develop anywhere along the gastrointestinal tract, the stomach is the most common site of formation^3^.

Bezoars are classified according to their composition and include lactobezoars, pharmacobezoars, trichobezoars, and phytobezoars, composed of milk products, medications, hair, and indigestible plant material, respectively^2^. In the case of phytobezoars, coagulation, and precipitation of tannic acid generates a gelatinous polymer that favors particle agglutination. Among the four types of bezoars, phytobezoars are the most common^2^. The polymerization and precipitation of tannins may contribute to the formation of a cohesive mass that promotes particle aggregation^2^. Diagnosis is usually established through imaging studies, which characteristically reveal a well-defined intraluminal mass with heterogeneous internal density^1^. 

Management strategies range from conservative medical therapy to invasive procedures. Surgical extraction through enterotomy and endoscopic interventions such as decompression or fragmentation are established options. However, less invasive approaches - including chemical dissolution with agents such as carbonated beverages (e.g., Coca Cola®) - are increasingly being employed. In this report, we describe a case of a giant bezoar successfully dissolved with an enzyme complex, highlighting a potential therapeutic alternative in situations where standard interventions may be ineffective^1^^,^^2^^-^^6^. 

## CASE REPORT

A 67-year-old man presented in April 2022 with diaphoresis and episodes of vomiting containing fibrous material. Six years earlier, he had been diagnosed with urothelial carcinoma and prostate adenocarcinoma, for which he underwent surgical treatment followed by adjuvant radiotherapy. Subsequently, he developed radiation-induced enteritis with radiular involvement. Two years after his initial oncologic diagnosis, an asymptomatic giant bezoar was identified. He was advised to consume large amounts of Coca Cola® for several days; however, no scheduled follow-up was performed to confirm resolution.

**Figure 1:**
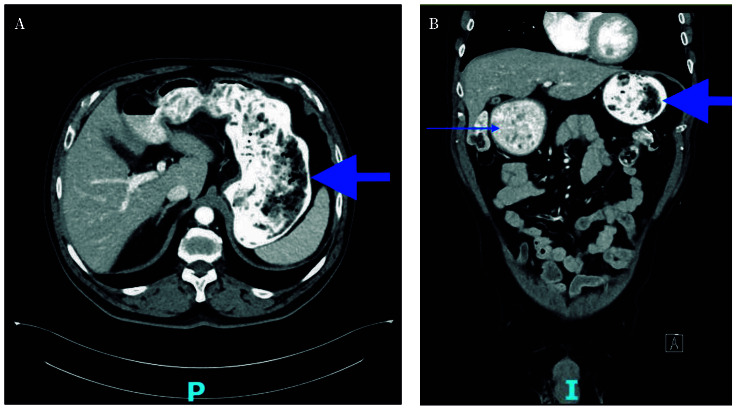
Computed tomography scan at admission. Axial (**A**) and coronal (**B**) view showing abundant, organized- appearing gastric content. Arrow: gastric chamber. Thin arrow: food retention at the pyloric antrum.

**Figure 2:**
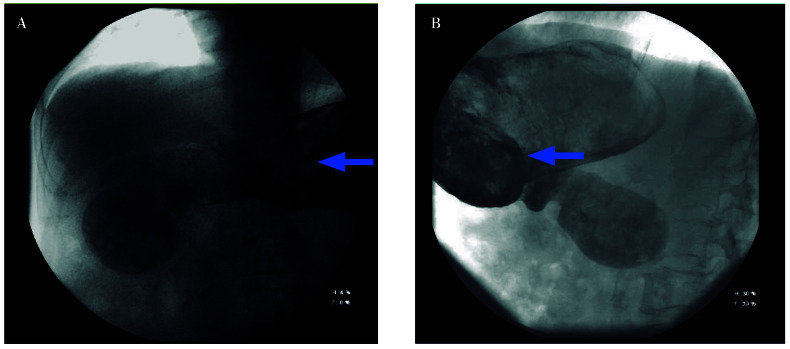
Pharyngo-esophageal study. Antero-posterior (**A**) and lateral (**B**) view where the stomach is dilated, containing heterogeneous contents that fill its entire lumen. The water-soluble contrast medium soaks the bezoar.

At the current admission, computed tomo-graphy (CT) demonstrated abundant, organized intragastric material consistent with retained food content (Fig. 1). The morphology was similar to that observed on previous CT examinations. These findings were suggestive of chronic gastric retention with impaired emptying at the level of the pyloric antrum. A pharyngoesophageal study revealed a large bezoar occupying the entire gastric cavity, with preserved esophageal motility (Fig. 2).

Based on previously published evidence^2^^,^^4^^,^^7^^-^^9^, treatment with an oral enzymatic complex (Digeston Plus®, HealthAid® House, Marlborough Hill, Harrow, Middlesex, UK), was initiated. The complex was administered once daily for three consecutive months. According to the manufacturer, each 310-mg tablet contains a combination of digestive enzymes and bacteria, including amylase (20,000 DU), protease (50,000 HUT), glucoamylase (45 AGU), pectinase (50 endo-PGU), alpha-galactosidase (225 GaIU), lactase (1,000 ALU), beta-glucanase (30 BGU), cellulase (1,000 CU), lipase CR (1,000 FIP), bromelain (125,000 FCCPU), xylanase (600 XU), hemicellulase (400 HCU), malt diastase (200 DP9), invertase (203 SU), papain (16,000 FCCPU), calcium chloride (120 mg), mint extract (20 mg), papaya extract (10 mg), pineapple extract (10 mg), *Lactobacillus acidophilus* (15 mg; 300 million CFU), and *Bifidobacterium longum* (15 mg; 300 million CFU).

**Figure 3:**
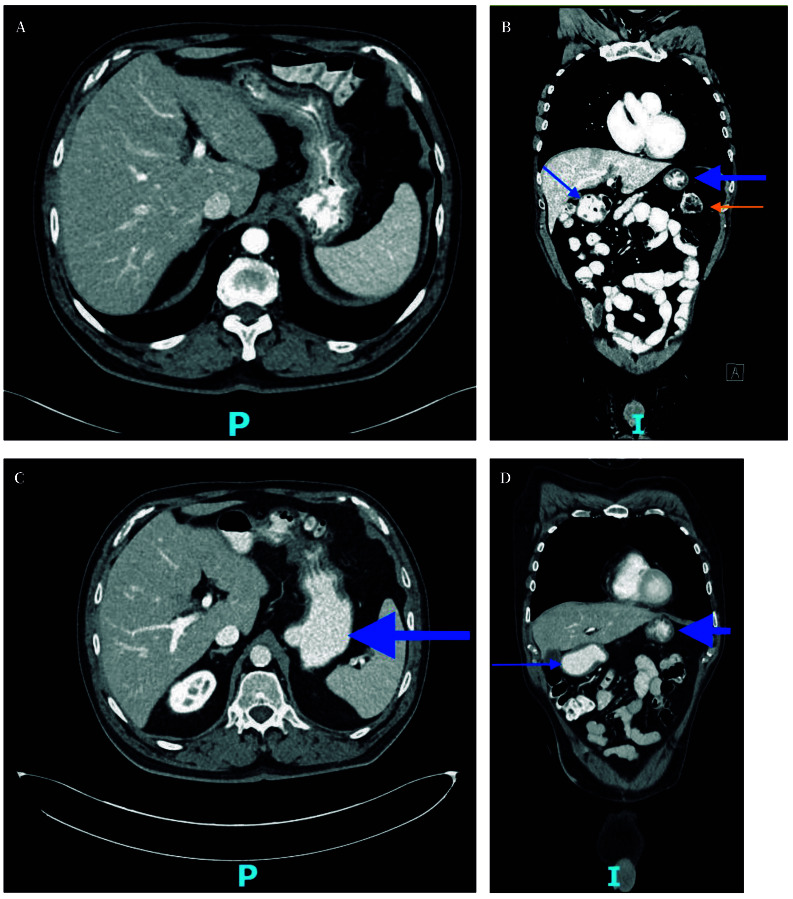
Computed tomography scan. Axial (**A**) and coronal (**B**) view at three month follow-up, with almost complete resolution of the gastric bezoar (blue arrow), with some organized-appearing content remaining in the antral region (thin blue arrow). Splenic colon flexure (orange arrow). Axial (**C**) and coronal (**D**) view at six month follow-up, with normal transit of oral contrast in gastric chamber (blue arrow) or pyloric antrum (thin blue arrow).

The patient experienced progressive clinical improvement, with complete resolution of symptoms after three months of therapy. Follow-up CT imaging performed at three months (Fig. 3A-B) and six months (Fig. 3C-D) demonstrated complete dissolution of the bezoar. 

Annual clinical follow-up was maintained through June 2025, with no evidence of symptom recurrence or bezoar reformation.

## DISCUSSION

Several risk factors for bezoar formation have been identified, including prior gastric surgery, Crohn disease, diabetes mellitus, dehydration, hypothyroidism, and renal failure, all of which may impair gastric motility and reduce gastric acidity^1^^,^^6^^,^^9^. In the present case, the patient had a history of diabetes mellitus and had undergone major abdominal oncologic surgery (radical cystectomy with ileal conduit urinary diversion) six years earlier. These factors may have contributed to altered gastrointestinal motility and chronic gastric stasis despite the absence of prior gastric resection.

Bezoars may remain asymptomatic or manifest with a range of gastrointestinal symptoms, including abdominal pain and postprandial fullness, depending on their location^1^^,^^6^^,^^9^. Gastric bezoars may cause mucosal ulceration with subsequent bleeding. Presentation as intestinal obstruction is uncommon, with small-bowel bezoars accounting for approximately 0.4%-4% of all cases of mechanical bowel obstruction^5^.

Diagnosis is established through endoscopic and/or radiologic evaluation. Upper gastrointestinal endoscopy plays a central role in both the detection and management of gastric bezoars. CT is particularly valuable in patients with suspected small-bowel bezoars, particularly when surgical intervention is being considered^1^^,^^5^^,^^6^. In the present case, abdominal CT scans enabled both confirmation of the initial diagnosis and objective follow-up of bezoar persistence and subsequent dissolution. This approach avoided repeated endoscopic procedures in a patient with a history of extensive oncologic surgery and prior abdominal irradiation.

Current treatment strategies for gastric phytobezoars include chemical dissolution, endoscopic fragmentation and extraction, and, in selected cases surgical removal. In contrast, intestinal bezoars are most commonly managed surgically, as they frequently present with small-bowel obstruction. Surgical intervention is generally reserved for cases in which the density and the consistency of the bezoar preclude noninvasive dissolution or endoscopic fragmentation. Recent studies have also described gastrointestinal decompression using a nasogastric tube or a long intestinal tube in patients presenting with acute small-bowel obstruction in the absence of signs of strangulation^1^^,^^3^^,^^5^.

Chemical dissolution represents an effective and less invasive alternative, particularly in patients with large enteral feed bezoars. The administration of isolated digestive enzymes constitutes one of the earliest conservative strategies reported in the literature.

Papain, a proteolytic enzyme derived from *Carica papaya*, has been used with reported success in small case series. Its mechanism of action involves degradation of the proteinaceous matrix that binds vegetable fibers within the bezoar^4^. However, its safety profile is unfavorable. Reports from the 1960s and 1970s describe serious adverse events, including gastric ulceration and esophageal perforation, which have substantially limited its use in current clinical practice^2^^,^^4^^,^^9^. Furthermore, subsequent experimental data suggest that its true lytic efficacy may be lower than initially reported when administered at dosages considered safe^2^.

Cellulase, which acts directly on cellulose - the principal structural component of phytobezoars - appears to offer an advantage in bezoar dissolution. Clinical series and case reports have documented high success rates in the treatment of gastric bezoars and, more selectively, in conservatively managed intestinal bezoars^4^^,^^9^. Nevertheless, its use presents practical limitations. In many countries, cellulase is not commercially available for oral administration or as a prescribed pharmaceutical formulation. In addition, treatment is not entirely devoid of risk, as cases of secondary intestinal obstruction resulting from distal migration of partially fragmented bezoars have been described^2^^,^^9^^,^^10^. For these reasons, close clinical and radiological monitoring was implemented in our patient. Serial imaging confirmed progressive dissolution of the bezoar, with no evidence of distal migration or recurrence during long-term follow-up.

Pancreatic enzyme extracts (amylase, lipase, and protease) have demonstrated utility primarily in bezoars of nutritional or esophageal origin, particularly those associated with casein-rich enteral feeding formulas^7^^,^^8^. Their effectiveness largely depends on concomitant alkalinization of the environment, which limits the extrapolation of these results to classical gastric phytobezoars. Moreover, the available evidence is restricted to isolated case reports and small case series, and no standardized treatment protocols have been established to date. In some patients, the administration of prokinetic agents has also been reported to be effective in resolving gastric bezoars^4^^,^^6^^-^^9^^,^^11^. 

Several studies have highlighted the utility of Coca Cola® for the dissolution of phytobezoars^11^. A systematic review by Ladas *et al*., reported that Coca Cola® administration, either alone or in combination with endoscopy, resulted in resolution in 91.3% of cases and was more effective than cellulose and papain. Nevertheless, the efficacy of Coca-Cola® is not uniform. Diospyrobezoars (typically associated with persimmon or pineapple ingestion) are characterized by increased hardness due to tannin polymerization and exhibit significantly lower response rates when used as initial therapy (23% compared with 60.6% for other phytobezoars)^11^. Although the underlying mechanism has not been fully elucidated, Coca-Cola® was ineffective in dissolving the bezoar in the present case, possibly due to its size, chronicity, or composition. This finding underscores the need to consider alternative conservative options in selected cases.

In addition, potential complications have been reported, including distal migration of partially dissolved fragments leading to secondary intestinal obstruction. This risk necessitates close clinical surveillance even after apparent endoscopic resolution^2^^,^^11^. Therefore, although Coca-Cola® represents an inexpensive, widely available, and generally safe therapeutic option, its effectiveness may be limited by the bezoar’s type, size, and consistency.

Endoscopy, both diagnostic and therapeutic, remains a cornerstone in the management of bezoars, either as a primary treatment modality or as an adjunct to dissolution therapies^2^. Techniques such as mechanical fragmentation, lithotripsy, and direct injection of dissolving agents have demonstrated high efficacy. However, these approaches often require prolonged procedures and multiple treatment sessions and are associated with an increased risk of complications, particularly in cases involving large bezoars or those with a hard consistency^7^.

In summary, the available literature indicates that no single conservative strategy is universally effective. Treatment selection should be individualized according to the type of bezoar, size, location, and consistency of the bezoar, as well as the experience of the treating center and medical team and the patient’s clinical characteristics. In this context, enzyme-based therapies - particularly those combining multiple mechanisms of action - represent a conceptually attractive alternative, as they simultaneously target different structural components of phytobezoars and may overcome some of the limitations observed with single-agent enzymatic therapies.

In our patient, the use of a multienzyme complex targeting different structural components of the phytobezoar was considered particularly appropriate, given the failure of single-agent chemical dissolution and the desire to avoid invasive procedures. Digeston Plus® is an enzyme complex that includes papain and cellulose in addition to pancreatic enzymes, resulting in a broad-spectrum formulation. As a commercially available product, its administration is feasible and relatively low cost compared with endoscopic or surgical interventions. The present case suggests that Digeston Plus® may represent a viable non-invasive option for the treatment of large gastric bezoars, particularly in cases in which Coca Cola® therapy is ineffective and invasive approaches are preferably avoided, even when bezoar size and location are associated with suboclussive symptoms.

In the present case, a gastric bezoar had already been identified on a follow-up abdominal CT scan performed two years after an oncologic surgery. At that time, the patient was asymptomatic, and oral contrast passed normally through all segments of the gastrointestinal tract. Abundant oral intake of Coca-Cola® was recommended both at that time and in the event of subsequent episodes of abdominal discomfort; however, the patient remained asymptomatic until presenting to our center with vomiting containing fibrous material.

Given the failure of Coca-Cola® to dissolve the phytobezoar, treatment with a multienzyme complex (Digeston Plus®) was initiated in an attempt to achieve non-invasive dissolution of the mass. In accordance with this therapeutic strategy, surgical intervention was avoided because of the patient’s history of oncologic surgery and radiotherapy, which increased the potential risk of surgical morbidity. Moreover, in the absence of acute complete obstruction or ischemia, a non-invasive dissolution-based approach was considered clinically appropriate in preference to endoscopic management. No adverse effects were observed during follow-up, and no recurrence has been detected to date.

This case illustrates the potential role of prolonged conservative management using a commercially available multienzyme complex in a patient with a giant gastric phytobezoar and significant comorbidities, in whom initial chemical dissolution with Coca-Cola® had failed. Although causal inference cannot be established from a single case, the favorable clinical course, radiological resolution, and long-term absence of recurrence, suggest that enzyme-based therapy may represent a feasible non-invasive option in carefully selected patients, particularly when endoscopic or surgical approaches carry increased risk.

This report should be regarded as hypothesis-generating and underscores the need for further prospective studies or well-designed case series to better define the efficacy, safety, and optimal indications of multienzyme complexes in the management of large gastric bezoars.

## Data Availability

Data supporting this study is available from the proprietary electronic health record system of Clínica Universidad de Navarra. Access to the data is subject to approval and a data sharing agreement due to legal and ethical restrictions, as the data contain confidential patient information and is only accessible to healthcare professionals directly involved in the patient’s care in accordance with applicable data protection regulations.
